# The Immunosuppressant Mycophenolic Acid Alters Nucleotide and Lipid Metabolism in an Intestinal Cell Model

**DOI:** 10.1038/srep45088

**Published:** 2017-03-22

**Authors:** Svenja Heischmann, Monika Dzieciatkowska, Kirk Hansen, Dieter Leibfritz, Uwe Christians

**Affiliations:** 1 Department of Anesthesiology, School of Medicine, University of Colorado Denver, Anschutz Medical Campus, Bioscience 2, 12705 East Montview Boulevard, Suite 200, Aurora, CO 80045, USA; 2 Department of Organic Chemistry and Instrumental Analytics, University of Bremen, Leobener Strasse NW2 C0041, 28359 Bremen, Germany; 3 Department of Biochemistry and Molecular Genetics, School of Medicine, University of Colorado Denver, Anschutz Medical Campus, Biological Mass Spectrometry Facility, 12801 East 17^th^ Avenue, Aurora, CO 80045, USA

## Abstract

The study objective was to elucidate the molecular mechanisms underlying the negative effects of mycophenolic acid (MPA) on human intestinal cells. Effects of MPA exposure and guanosine supplementation on nucleotide concentrations in LS180 cells were assessed using liquid chromatography-mass spectrometry. Proteomics analysis was carried out using stable isotope labeling by amino acids in cell culture combined with gel-based liquid chromatography-mass spectrometry and lipidome analysis using ^1^H nuclear magnetic resonance spectroscopy. Despite supplementation, depletion of guanosine nucleotides (p < 0.001 at 24 and 72 h; 5, 100, and 250 μM MPA) and upregulation of uridine and cytidine nucleotides (p < 0.001 at 24 h; 5 μM MPA) occurred after exposure to MPA. MPA significantly altered 35 proteins mainly related to nucleotide-dependent processes and lipid metabolism. Cross-reference with previous studies of MPA-associated protein changes widely corroborated these results, but showed differences that may be model- and/or method-dependent. MPA exposure increased intracellular concentrations of fatty acids, cholesterol, and phosphatidylcholine (p < 0.01 at 72 h; 100 μM MPA) which corresponded to the changes in lipid-metabolizing proteins. MPA affected intracellular nucleotide levels, nucleotide-dependent processes, expression of structural proteins, fatty acid and lipid metabolism in LS180 cells. These changes may compromise intestinal membrane integrity and contribute to gastrointestinal toxicity.

The anti-proliferative drug mycophenolic acid (MPA) is a cornerstone of most immunosuppressive regimens after solid organ transplantation^1^. MPA exerts its immunosuppressive properties by inhibition of inosine 5′-monophosphate dehydrogenase (IMPDH), the enzyme that limits *de novo* purine synthesis. Most cell types are able to replenish purine pools *via* a salvage and a *de novo* pathway. Lymphocytes, however, are almost fully dependent on purine *de novo* synthesis. As clonal expansion of lymphocytes is essential for an immune response, MPA inhibits precursor generation for deoxyribonucleic acid synthesis and consecutively lymphocyte proliferation, thereby effectively blocking immunoreactions. Nevertheless, gastrointestinal (GI) intolerability limits applicability of MPA-based regimens^2^,^3^,^4^,^5^,^6^. GI side effects are the main reason for dose changes or discontinuation, which often jeopardize short- and long-term outcomes of graft survival^4^,^5^,^7^,^8^,^9^,^10^. Abdominal pain, diarrhea, mucosal changes such as ulcers, and submucosal inflammation are common signs of MPA GI toxicity; similarities to Crohn’s disease have been demonstrated^11^. In individuals suffering from Crohn’s disease, bacterial invasion of the epithelium^12^ due to a compromised mucous layer and/or epithelial barrier triggers an inflammatory cascade^11^, which leads to the aforementioned symptoms.

The etiology of MPA-related GI adverse effects is not yet fully understood and the underlying molecular mechanisms have never comprehensively been studied. However, several hypotheses exist regarding the origin of MPA’s adverse effects on the GI tract. It has been suggested that the main mediators of toxicity are MPA’s acyl glucuronide metabolite (AcMPAG) and eventually the morpholino ester moiety N-(2-hydroxyethyl) morpholine, which is cleaved from the prodrug mycophenolate mofetil (MMF) to result in the active MPA^13^. AcMPAG can form protein adducts^13^,^14^, N-(2-hydroxyethyl) morpholine may cause local irritation of the epithelium^13^. Moreover, it has been hypothesized that MPA promotes inflammation by proliferation inhibition of the GI tract’s rapidly dividing epithelial cells^6^. This may lead to disruption of the GI barrier^13^ and Crohn’s disease-like symptoms. This hypothesis has been challenged as purines, released during the ingestion of cells from dietary sources, are highly abundant in the GI lumen^13^. Purines can enter the cell lumen *via* passive diffusion, by utilization of a transporter for nucleotides, or *via* a carrier-mediated process^13^.

Toxicodynamic mechanisms are not necessarily linked to a drug’s mechanism of action. Therefore, we investigated mechanisms of MPA toxicity at the cellular level in the presence of extracellular guanosine as provided under physiologic conditions in the intestinal human colon adenocarcinoma cell line LS180 by untargeted analysis of protein and metabolite changes. Compared to an *in vivo* system, a cellular system allows control of local drug and guanosine concentrations while avoiding secondary pathological processes, such as inflammation, which may interfere with the assessment of the underlying toxicity mechanisms in an *in vivo* setting. Human epithelial-like colon cancer LS180 cells were chosen based on a comprehensive literature review of available models applicable to elucidate adverse effects of drug treatment on the GI tract. Most importantly, LS180 cells express the pregnane X receptor, and are therefore able to upregulate/induce the expression of drug metabolizing enzymes as seen *in vivo*^15^.

## Materials and Methods

### Cell culture model

LS180 cells (ATCC, Manassas, VA; CL-187, passage number 40) were cultured and supplemented with guanosine (up to 1 mM) during MPA exposure (up to 250 μM).

### Measurement of high-energy phosphate levels

Following extraction with perchloric acid, concentrations of the high-energy phosphates adenosine, guanosine, uridine, and cytidine tri-, di- and monophosphates (ATP, ADP, AMP, GTP, GDP, GMP, UTP, UDP, UMP, CTP, CDP, CMP) and the cofactors nicotinamide dinucleotide (NAD^+^), NAD^+^ phosphate (NADP^+^), and flavin adenine dinucleotide (FAD) were measured on an API 4000 triple quadrupole (AB Sciex, Concord, ON, Canada) by high-performance liquid chromatography-mass spectrometry (HPLC-MS)^16^.

### SILAC, GeLC-MS, and analysis of differentially expressed proteins

Cells were labeled with heavy lysine ([U-^13^C_6_]-L-lysine) or light lysine ([^12^C_6_]-L-lysine) using Stable Isotope Labeling by Amino Acids in Cell Culture (SILAC, Invitrogen, Carlsbad, CA). Cells were lysed and after 1D gel electrophoresis, bands were digested with trypsin. HPLC-MS analysis was performed on an LTQ-FT Ultra hybrid mass spectrometer (Thermo Scientific, San Jose, CA). Gel-based liquid chromatography-mass spectrometry (GeLC-MS) data were analyzed using the MaxQuant software package^17^ (Max Planck Institute of Biochemistry, Martinsried, Germany, version 1.2.2.5), the Database for Annotation, Visualization, and Integrated Discovery (DAVID)^18^,^19^ and Pathway Palette^20^.

### Cross reference with proteome alterations in rat models and other human cell lines of non-cancerous origin

For comparison of data collected using the LS180 cell model (human colon cancer) with those collected using common rat models (Wistar rats) proteins/genes from the three available publications on this topic (kidney tissue of MMF-treated rats^14^, liver and colon tissue of MMF-treated rats^21^, analysis of gene expression (complementary deoxyribonucleic acid microarray analysis) in liver and gut of MMF-treated rats^22^) were pooled (Supplementary Table S1). For comparison with data collected through models using other human cell lines (HEK-293, CCRF-CEM; differential proteome analysis) protein/gene data from two available publications were pooled^23^,^24^ (Supplementary Table S2). DAVID software was used for comparison.

### NMR spectroscopy analysis

Nuclear Magnetic Resonance (NMR) spectra were recorded on a Bruker DRX 600 spectrometer (Bremen, Germany) and processed using MestRe-C software (version 4.9.9.9, Mestrelab Research, Santiago de Compostela, Spain). Compounds were identified by comparison of signals from ^1^H-^13^C HSQC NMR spectra to database values^25^,^26^.

### All experimental procedures

For additional information and details please see the Supplementary Materials and Methods.

### Statistics

Statistical significance of differences between groups was determined by one-way analysis of variance (ANOVA) combined with Scheffe’s *post*-*hoc* test. GeLC-MS data were analyzed by online tools using Benjamini and/or Bonferroni correction for multiple testing.

## Results

### Levels of nucleotides, cofactors, and nucleotide energy charges in LS180 cells after exposure to MPA

Although intestinal cells are known to import nucleotides from the GI lumen^13^, we hypothesized that MPA treatment compromises intracellular nucleotide concentrations of LS180 cells. Significant impairment of intracellular nucleotide levels despite guanosine supplementation was observed in our cell culture model (Table 1; Supplementary Figs S1 and S2). While supplementation with 200 μM and 1 mM guanosine largely restored adenosine nucleotide concentrations in LS180 cells treated with 250 μM MPA (Supplementary Fig. S1), guanosine nucleotide concentrations remained significantly lower than in controls not treated with guanosine (p < 0.001) for MPA concentrations ≥5 μM despite guanosine supplementation for 24 and 72 h (Supplementary Fig. S1). Even for LS180 cells treated with 1 mM exogenous guanosine; GTP, GDP, and GMP levels were only approximately 30% of the controls. Addition of guanosine to culture media reversed changes in UMP and UDP levels after 24 and 72 h that were observed without guanosine supplementation (Supplementary Fig. S1). UTP levels, on the other hand, were significantly higher (p < 0.01 and 0.001, respectively) after exposure to MPA concentrations ≥5 μM and in the presence of 200 μM and 1 mM guanosine after 72 h. In most cases, changes in cytidine nucleotide levels were reversed by supplementation with guanosine (Supplementary Fig. S2). Slightly elevated values suggest imbalances in cytidine nucleotide levels in LS180 cells despite of, or possibly due to, guanosine supplementation of the culture media. Disturbances in NAD^+^, NADP^+^, and FAD concentrations, which mainly occurred after 72 h of exposure to higher MPA concentrations in the absence of guanosine supplementation, were similar to control levels when incubation media were supplemented with guanosine (Supplementary Fig. 2). While nucleotide energy charges in MPA-treated cells did not show marked differences compared to control cells with guanosine supplementation <1 mM, uridylate energy charges after 72 h of treatment with 250 μM MPA and 1 mM guanosine increased remarkably (125.3 ± 5.0% of controls, p < 0.05, Supplementary Fig. S2). In summary, some negative effects of MPA on nucleotide levels were partially or fully reversed by supplementation with guanosine. Despite supplementation with 1 mM guanosine, levels of all guanosine nucleotides were significantly decreased (p < 0.001) and levels of uridine and cytidine nucleotides were upregulated with consequent effects on the uridylate energy charge. Levels of the second messenger cyclic adenosine monophosphate and cyclic guanosine monophosphate were mostly unaffected by MPA, regardless of exogenous guanosine concentrations (Supplementary Fig. 3), when examined by enzyme-linked immunosorbent assays.

### Investigation of proteome alterations in LS180 cells after exposure to MPA and cross reference of results with proteome alterations in rat models and other human cell lines of non-cancerous origin

Expression levels of 35 proteins were significantly changed (p < 0.05, >1.2-fold) in MPA-treated LS180 cells despite supplementation with 1 mM guanosine as identified by SILAC in combination with GeLC-MS (Table 2). As a representative example, HPLC-MS data for the VIILMDPFDDDLK peptide, one of the unique peptides that served to identify the protein long-chain acyl-coenzyme A synthetase 5 (ACSL5) is shown in Supplementary Fig. S4). Functional annotation clustering of the 35 affected proteins using DAVID software yielded the four annotation clusters “guanyl nucleotide-binding”, “lipid catabolic process”, “protein polymerization”, and “mitochondrial membrane” (enrichment scores >2.7, Supplementary Table S3). Analysis using additional gene ontology tools repeatedly indicated links to purine/lipid metabolism as well as the subcellular compartments mitochondria and cytoskeleton (functional annotation chart, pathway enrichment analysis, and functional annotation classification; please see Supplementary Tables S4–S6, respectively).

Affected proteins were mapped to KEGG pathways (Supplementary Figs S5 and S6) illustrating dysregulation of fatty acid degradation and synthesis during MPA exposure (Supplementary Figs S5 and S6). DAVID analysis of data from rat models pooled form the literature^14^,^21^,^22^ showed effects of MPA on “carbohydrate catabolic processes” and “nucleotide/purine nucleotide metabolic processes” as well as on pathways of carbohydrate and amino acid metabolism (functional annotation clustering and pathway enrichment analysis, please see Supplementary Tables S7 and S8, respectively). Analysis of proteins/genes that were affected by MPA treatment in other human cell types (HEK-293 and CCRF-CEM^26^,^27^) did not yield any significantly enriched annotation terms or pathways.

**Figure 1:**
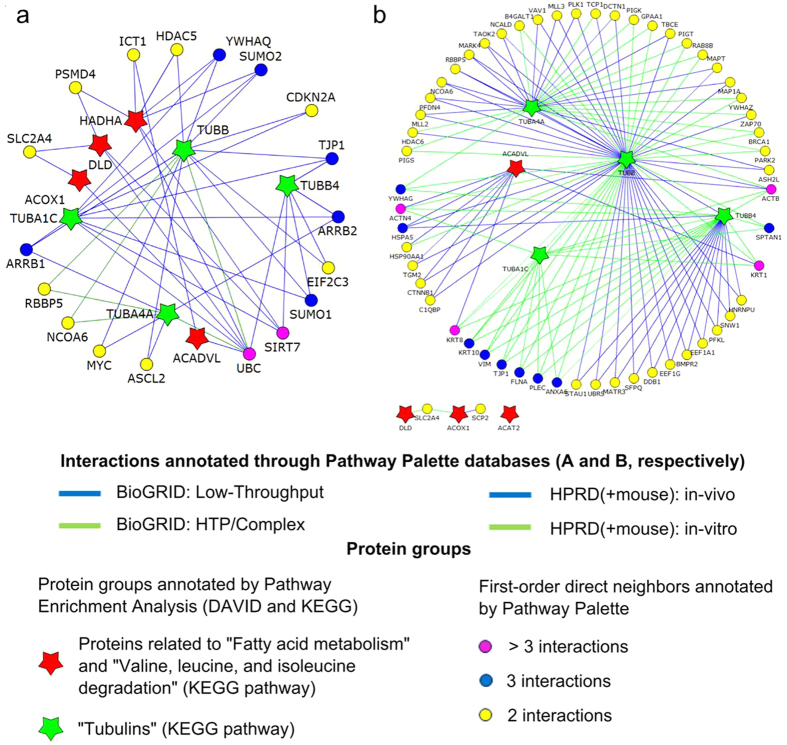
Network of proteins of enriched pathways and first-order shared neighbors constructed using the Pathway Palette software. (**a**) Network constructed using the BioGRID database. (**b**) Network constructed using the HPRD database. First-order shared neighbors were assigned based on interaction data in the databases. Interactions between interconnecting proteins are not shown. 18 (BioGRID) and 57 (HPRD) protein pairs linked through first-order shared neighbors and 0 (BioGRID) and 3 (HPRD) direct interactions between the proteins of affected pathways were found. Star-shaped nodes represent proteins found in significantly affected pathways annotated through Pathway Enrichment Analysis using DAVID and KEGG with a color scheme corresponding to pathways as classified in the legend and Supplementary Table S1. Proteins are indicated by their gene names. Proteins with ≥3 interactions are listed in Supplementary Table S2 including abbreviation, gene name accession number, and physiological function.

A protein-protein interaction network was generated from the 13 proteins of significantly enriched KEGG functional categories (EASE score <0.5), i.e. ACSL5, acetyl-coenzyme A acetyltransferase (ACAT2), very long-chain specific acyl-coenzyme A dehydrogenase (VLCAD), peroxisomal acyl-coenzyme A oxidase 1 (AOX), trifunctional enzyme subunit α (TFP), acetyl-coenzyme A acyltransferase (ACAA2), dihydrolipoamide dehydrogenase (DLD), tubulin α-1C chain (TUBA1C), tubulin α-4A chain (TUBA4A), tubulin β-4A chain (TUBB4A), tubulin β chain (TUBB), succinyl-coenzyme A ligase [ADP/GDP-forming] subunit α (SCS-α), and fatty acid-binding protein 1 (FABP1) with the purpose of identifying additional potentially affected proteins. The protein-protein interaction network generated based on the BioGrid database (Fig. 1, Panel a) and Human Protein Reference Database (Fig. 1, Panel b) revealed 18 and 55 first-order direct neighbors with more than two interactions, respectively. First-order direct neighbors were sorted based on number of interactions with differentially expressed proteins (Supplementary Table S9). Candidate proteins to be affected by MPA (chosen based on biological/metabolic proximity to differentially expressed proteins and/or interactions with multiple differentially expressed proteins) were interrogated by Western blot analysis, but no differences in protein expression for representative proteins were found.

### Western blot analysis

**Figure 2:**
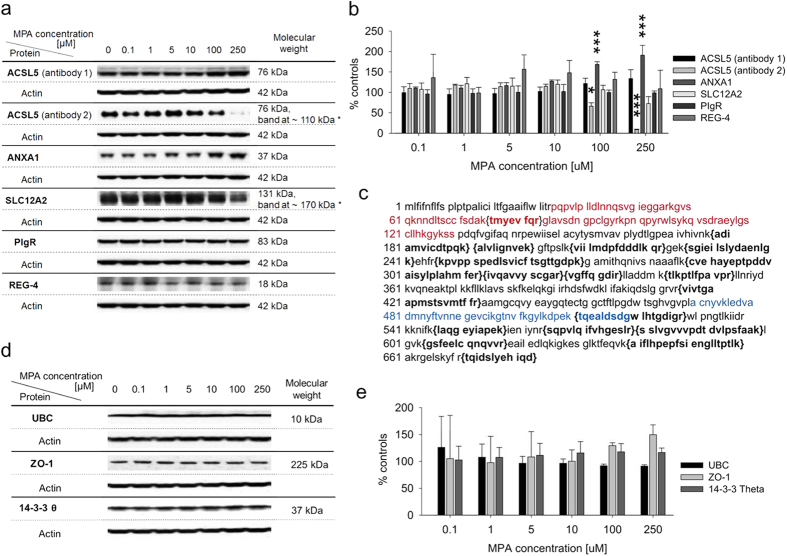
Interrogation of SILAC GeLC-MS results by Western blot analysis. (**a**) Western blots for interrogation of results of SILAC GeLC-MS experiments (n = 3; *difference between actual and predicted band size potentially due to post-translational modifications, post-translational cleavage, splice variants, relative charge, or multimerization). (**b**) Relative intensities of bands normalized to β-actin and statistical significance of changes determined by one-way ANOVA combined with Scheffe’s *post*-*hoc* test with *p < 0.05, ***p < 0.001. (**c**) Amino acid sequence of the NP 976313.1 variant of isoform b (683 aa protein) of ACSL5. 18 unique peptides identifying ACSL5 in the SILAC GeLC-MS experiment by Mascot are marked (bold, in brackets) as well as immunogen sequences of anti-ACSL5 antibody 1 (Sigma, WH0051703M1; red) and 2 (Abcam, ab104892; blue) to elucidate the discrepancy of MS and western blot results. (**d**) Western blots of potentially affected proteins as identified by Pathway Palette analysis (n = 3). (**e**) Comparison of relative intensities of bands normalized to β-actin did not show significant changes in protein expression.

Western blot analysis was carried out for five representative proteins to verify SILAC GeLC-MS results, namely ACSL5 (increased), annexin A1 (ANXA1; increased), solute carrier family 12 member 2 (SLC12A2, decreased), polymeric immunoglobulin receptor (PIgR, decreased), and regenerating islet-derived protein 4 (REG-4; decreased; Fig. 2, Panel a and b), but SILAC results could only partially be confirmed. Two western blots for ACSL5, conducted with different antibodies raised against different epitopes, showed differential ACSL5 expression. While analysis using the first antibody (Sigma, WH0051703M1) showed no significant change for ACSL5 expression for MPA-treated LS180 cells, analysis using the second antibody (Abcam, ab104892) showed significant decreases of ACSL5 for treatment with 100 μM (p < 0.05) and 250 μM (p < 0.001) MPA. To illustrate these findings and visualize the discrepancy between MS and western blot results as well as western blot results using the two different antibodies, the ACSL5 amino acid sequence is listed in Fig. 2, Panel c; unique peptides and epitopes are indicated.

In addition, levels of protein expression were investigated by western blot analysis for three representative proteins identified by Pathway Palette analysis as first-order direct neighbors and therefore potentially affected due to metabolic proximity to altered proteins as identified by SILAC and GeLC-MS, i.e. tight junction protein ZO-1 (ZO-1), 14-3-3 protein θ (14-3-3 θ; simultaneous identification by BioGRID and Human Protein Reference Database (HPRD) databases), and polyubiquitin-C (UBC; 9 interactions assigned by BioGRID). No differences in expression levels of these proteins were found (Fig. 2, Panel d and e).

### Quantitative ^1^H-NMR spectroscopy of lipid extracts of LS180 cell

**Figure 3:**
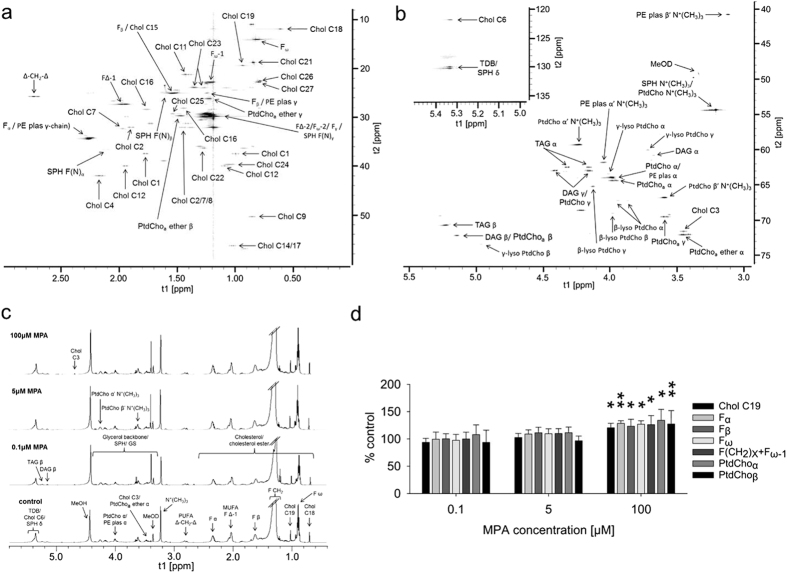
NMR spectra of a lipophilic LS180 cell extract. (**a**) ppm region 0.0–0.3 (t1)/10–60 (t2) of a ^1^H-^13^C HSQC spectrum. (**b**) ppm regions 3.0–5.5 (t1)/40–75 (t2) and 5.0–5.5 (t1)/120–135 (t2) of a ^1^H-^13^C HSQC spectrum. (**c**) ^1^H-NMR spectra of lipophilic LS180 cell extracts. Compounds were identified based on databases^25^,^26^. Cells were incubated with different MPA concentrations and 1 mM guanosine for 72 h (dosed every 24 h). Amino acid abbreviations are based on IUPAC nomenclature, Chol: cholesterol/cholesterolester, DAG: diacylglycerols, F: fatty acid, Fα/β: carbon atom in α/β-position to carbonyl carbon atom, FΔ: carbon atom at a double bond, Fω: terminal carbon atom, MUFA: monounsaturated fatty acids, R-N+ (CH_3_)_3_: trimethyl ammonium compounds, PE: phosphatidylethanolamine, plas: plasmalogen, PtdCho: phosphatidylcholine, PUFA: polyunsaturated fatty acids, SPH: sphingomyelin, TAG: triacylglycerols, TDB: total number of double bonds (MUFA+PUFA). (**d**) Changes in concentrations of cholesterol, selected fatty acid carbon positions, and phosphatidylcholine calculated from ^1^H NMR spectra of lipophilic LS180 cell extracts (n = 6). Significance was determined by one-way ANOVA combined with Scheffe’s *post*-*hoc* test with *p < 0.05, **p < 0.01 *vs*. controls.

After confirmation of signal assignments by 2D NMR experiments (Fig. 3, Panel a, b, and c), quantification of abundant compounds revealed increases (p < 0.05 or p < 0.01) in cholesterol/cholesterol esters (Chol C19), fatty acids (F_α_, F_β_, F_ω_, F(CH_2_)_x_ + _Fω−1_), and phosphatidylcholines (PtdCho α/β), after exposure to 100 μM MPA and 1 mM guanosine supplementation (Fig. 3, Panel d). Trimethylammonium compounds/choline head groups (N^+^(CH_3_)_3_), diacylglycerols (DAG β), riacylglycerols (TAG β), carbon atoms at a double bond (FΔ-1, monounsaturated fatty acids (MUFA)), polyunsaturated fatty acids (FΔ-CH2-Δ, PUFA), and total number of double bonds (TDB, MUFA+PUFA) were unchanged.

## Discussion

The most important molecular mechanisms of mycophenolate intestinal toxicity described in the literature, as of today, are limited to the covalent binding of the acyl glucuronide metabolite of MPA (AcMPAG) to various intestinal proteins^21^ and the disruption of tight junctions^27^. Two follow up studies showed that the disruption of intestinal tight junctions was associated with chromatin histone modifications, including an increase in midkine concentrations^28^,^29^. In contrast to these targeted studies, as aforementioned, in the present study, we took a comprehensive, non-targeted, unique combined proteo-metabolomic approach that resulted in a much more complete picture of the time- and dose-dependent effects of mycophenolic acid on intestinal cell protein concentrations and cell metabolism at various levels of guanosine supplementation.

The gastrointestinal epithelium is locally exposed to high MPA concentrations, especially at the locations where MMF/MPA is released from the formulation, which are in the range of the concentrations tested in the present study. Other studies used MPA at lower concentrations (e.g. 0.1–5 μM^30^, 10 μM^31^) *in vitro* than the present study and guanosine concentrations necessary to reverse MPA-induced effects were around 50–100 μM^30^,^31^. Such studies evaluated inhibition of cell proliferation, which depends on availability of guanosine nucleotides, but does not necessarily reflect levels of intracellular guanosine pools and the concentrations present at the intestinal epithelium. Due to the differences in MPA and guanosine concentrations, our results cannot directly be compared to such previous studies. As in the present study supplementation even with concentrations as high as 1 mM guanosine could not restore guanosine nucleotide levels, it is likely that a similar dysregulation of nucleotides occurs intracellularly *in vivo*. In particular GDP-, UDP-, and CDP-linked intermediates play a key role in lipid metabolism, membrane synthesis, and protein glycosylation^32^,^33^, suggesting negative effects of MPA on epithelial barriers.

Binding of guanosine nucleotides was associated with the function of eight of the 35 differentially expressed proteins in LS180 cells (Supplementary Table S4, #1: “guanyl nucleotide-binding”). Effects of MPA have been attributed to intracellular guanosine nucleotide depletion^34^,^35^,^36^,^37^, e.g. changes in protein glycosylation especially studied with respect to modifications of adhesion molecules^36^,^37^. Nevertheless, the notion that intracellular guanosine nucleotides in GI epithelial cells are abundant due to import of guanosine from the intestinal lumen so that MPA-mediated inhibition of *de novo* synthesis of GTP has little or no effect^13^, is not confirmed by the present study.

Effects of MPA and its metabolite AcMPAG on tubulin polymerization (even in the presence of exogenous GTP) have been described by Feichtinger *et al*.^37^. Effects on tubulins were also seen in LS180 cells; tubulin subunits constitute 50% of proteins of group #1 (“guanyl nucleotide-binding”) and 100% of proteins of group #3 (“protein polymerization”). Interestingly, potentially affected proteins identified by Pathway Palette are also linked to enriched functions/annotation terms (i.e. regulation of cell cycle, signaling processes, transcription, tight junctions, cytoskeletal properties, and transport; Supplementary Table S3).

Feichtinger *et al*.^37^ reported induction of tubulin polymerization, while in the present study the cellular amount of four tubulin subunits was decreased to 74.0–79.2% of control values in LS180 cells treated with 250 μM MPA (Table 2). Morath *et al*., on the other hand, found cytoskeletal proteins (vinculin, tubulin) to be downregulated in human fibroblasts, suggesting that MPA exposure results in cytoskeletal dysfunction^15^. Literature on MPA’s effect on lipid and fatty acid metabolism is scarce^38^. However, clinical studies show association of MMF and hyperlipidemia^38^, which is in accordance with our findings that expression of four proteins attributed to the term “Lipid catabolic process” (Supplementary Table S3) were significantly increased. Conversely, no significant changes occurred in serum cholesterol or triglyceride levels of rabbits on high-cholesterol diets with or without MMF in a study by Subramanian *et al*.^38^.

Data from studies involving LS180 cells differ from corresponding rat model data (Supplementary Table S5 *vs*. S8). These differences are likely due to differences in model systems (*in vitro vs. in vivo*), species (human *vs*. rat), doses, platforms, and data analysis methods. Comparing proteomics data in the present study with data of MMF treatment of rats (#1 in Supplementary Table S5 *vs*. #6 in Supplementary Table S8), four proteins involved in fatty acid metabolism were affected in rats, but Functional Annotation Clustering of rat model data (Supplementary Table S7) yielded only clusters related to carbohydrate or nucleotide processes. Sufficient data on MPA’s effects on glucose metabolism is lacking^38^. No effects of MMF on glucose metabolism were observed in clinical trials and effects of MMF on insulin secretion and insulin gene expression seem to differ between species *in vitro* (MMF inhibits insulin secretion in rat islets^39^, but does not show effects on insulin secretion or insulin gene expression in human islets^40^). Although multiple other factors such as the cancerous nature of LS180 cells, methodological differences, and sample size may have an effect, this pattern can be traced when comparing the present results from the LS180 cell model with *in vivo* data from rat models. As shown in Supplementary Table S7 (rat), the term “carbohydrate catabolic process” is listed as the most enriched term, but is not listed among Functional Annotation Clusters from analysis of LS180 cell data (Supplementary Table S3). Pathway Enrichment Analyses of 35 proteins in LS180 cells affected by MPA exposure and 76 proteins/genes affected by MPA exposure in rats (Supplementary Table S3) show the same model-dependent pattern using the KEGG database and DAVID’s Functional Annotation Chart tool (Supplementary Table S5 *vs*. S8). For LS180 cells, several of the listed terms were linked to lipid metabolism (e.g. #1: fatty acid metabolism, #5: propanoate metabolism, #6: fatty acid elongation in mitochondria, #7: peroxisome proliferator-activated receptor (PPAR) signaling pathway; Supplementary Figs S5 and S6). Analysis of rat proteome data revealed several enriched terms linked to lipid metabolism (#4: propanoate metabolism, #6: fatty acid metabolism, #11: butanoate metabolism). On the other hand, alterations in the expression of proteins involved in glucose and protein metabolism/amino acid degradation were primarily observed in rats undergoing MMF treatment (i.e. #1: glycolysis/gluconeogenesis, #2: pyruvate metabolism, #3: arginine and proline metabolism, #7: tryptophan metabolism, #8: phenylalanine metabolism, #10: fructose and mannose metabolism), while only two pathways involved in amino acid degradation were significantly affected in LS180 cells (i.e. #2: valine, leucine, and isoleucine degradation, #5: propanoate metabolism).

To corroborate an increase in ACSL5 observed in the SILAC proteomics experiment after exposure of LS180 cells to MPA, samples were also analyzed using western blot. At first only a small, non-significant change in expression levels was found. Nevertheless, repeat analysis using a different antibody showed a marked decrease in ACSL5 protein levels after exposure to 100 μM and 250 μM MPA (66.4 ± 8.3% and 8.7 ± 1.2% of controls). Only unmodified peptides and standard variable modifications were included for protein quantification in MaxQuant. The approximately 2-fold increase in ACSL5 assessed in SILAC GeLC-MS experiments related to these unmodified peptides. The decrease seen in western blots using the second anti-ACSL5 antibody was likely due to a modification of the antibody binding site (marked in blue in the ACSL5 amino acid sequence in Fig. 2, Panel c), as this site is not part of a unique peptide that served for protein identification (marked in bold and brackets in Fig. 2, Panel c).

Moreover, compared to controls, UTP and GTP levels were dysregulated in MPA-treated LS180 cells despite supplementation with 1 mM guanosine. Nucleotides are intermediates in the glycosylation of proteins and lipids^41^,^42^: glucose, galactose, and various amines are transferred to proteins *via* UDP intermediates, fucose and mannose are transferred *via* GDP. Although MPA induces inhibition of glycosylation of proteins through depletion of guanosine nucleotides^12^,^43^, augmentation of glycosylation in LS180 cells may occur due to increased UTP (Supplementary Fig. S1).

While increases in ANXA1 expression during MPA exposure as observed in the SILAC proteomics analysis could be verified by western blot (Fig. 2, Panel a and b), no changes in SLC12A2, PIgR, and REG-4 expression were detected by western blot. Nevertheless, for the following reasons, this does not preclude the SILAC GeLC-MS results from being correct. As discussed for the ACSL5 western blot results, the generally semi-quantitative nature of western blot analyses and MPA’s known ability to strongly influence protein glycosylation may have compromised the western blot analysis. Moreover, the results from nucleotide HPLC-MS, DAVID, and NMR-based metabolomics analyses and the literature^22^ support that MPA indeed affects SLC12A2 and PIgR expression.

The majority of affected proteins (Table 2) is involved in lipid metabolism. Proteins of “Fatty acid metabolism”, “Fatty acid elongation in mitochondria” (Supplementary Table S5), and “Lipid catabolic processes” (Supplementary Table S3 and S6) were significantly upregulated in LS180 cells after MPA treatment (with the exemption of ACAT2, which was downregulated). The term “mitochondrial membrane” (Supplementary Table S3) lists almost exclusively proteins that were found to be increased (with the exemption of SCS-α, which was downregulated). These results also suggested imbalances in membrane composition.

To further examine the impact of changes in the expression of proteins involved in lipid metabolism, intra- cellular lipid patterns were assessed using an ^1^H-NMR-based metabolomics approach. Cholesterol is a precursor for signaling molecules and is a fundamental constituent of cell membranes^44^ and changes of its intracellular levels may mediate drug toxicity. With the observed increases in phosphatidylcholines and cholesterol levels, homeostasis of two major membrane constituents is affected in LS180 cells exposed to MPA. In fact, phosphatidylcholines are the most prominent membrane phospholipids and crucial for maintenance of GI barrier function^45^. Intracellular phosphatidylcholines are secreted by epithelial cells and passaged across tight junctions into the apical mucus layer^46^, where they contribute to the establishment of a hydrophobic surface of the colonic mucus layer^45^. In the plasma membrane of enterocytes, phosphatidylcholine modulates the mucosal signaling state as lipid composition of membranes is a regulatory parameter of inflammatory responses^45^. Decreased levels of luminal phosphatidylcholines in colonic mucus have been linked repeatedly to ulcerative colitis, an inflammatory bowel disease similar to Crohn’s disease^45^,^46^, but this finding does not apply to Crohn’s disease patients^47^,^48^. Upregulation of intracellular phosphatidylcholines in MPA-treated LS180 cells suggests disturbances of lipid levels linked to mucosal defense. Phosphatidylcholine is synthesized from choline *via* the Kennedy-pathway involving cytidine nucleotides^49^. Phosphocholine and CTP are formed from CDP-choline and pyrophosphate, consecutively CDP-choline and diacylglycerol (or alkyl-acylglycerol) are converted to phosphatidylcholine (with CMP as byproduct). Slightly elevated levels of cytidine nucleotides observed at 72 h with 100 μM MPA and 1 mM guanosine (Supplementary Fig. S2, Table 1) may be associated with increased phosphatidylcholine biosynthesis. Furthermore, increases in fatty acid, diacylglycerol, and triacylglycerol may also be related to impaired composition of membrane lipids, e.g. other phospholipids than phosphatidylcholines, which constitute membranes^50^.

The major limitation of this study is the use of a cancer cell line and the associated potential differences in metabolism, protein expression and their regulation compared to normal human intestinal cells. Nevertheless, as aforementioned, the results of the present study were cross-referenced to currently available proteomics data on MPA toxicity in rat models and other human cell culture models using cells of non-cancerous origin using DAVID (pathway enrichment analysis using KEGG). Overlap of the results with those of the present study support the validity of the LS 180 cell model. Although it was not our objective to validate the LS180 cells culture model, the results of the present study are further proof that LS180 cells are an attractive model as a fast and simple screen for potential intestinal toxicity of drugs and drug candidates beyond MPA and are an alternative to more cumbersome but widely used Caco-2 cell models. An interesting question is if, and how, the observed proteomics and metabolomics changes are associated with impairment of the intestinal barrier. The present study was not designed to assess this question and this will be evaluated in a follow up.

In conclusion, the present study based on the LS180 cell model and a metabolomics-proteomics profiling strategy suggests that MPA-induced GI disturbances involve the dysregulation of nucleotide-dependent processes and lipid metabolism. Our data support that MPA’s GI toxicity is linked to the drug’s mechanism of action resulting in disturbance of intracellular guanosine levels, that are important not only for cell proliferation but also other vital intracellular processes. Importantly, the results of the present study suggested that, other than hypothesized, guanosine supplementation does not fully reverse the negative effects of MPA on nucleotide metabolism. The negative effects of MPA in the present study were concentration-dependent suggesting that the avoidance of high local concentrations in the intestine, for example by the development of sustained release formulations, may improve its GI tolerability.

## Additional Information

**How to cite this article:** Heischmann, S. *et al*. The Immunosuppressant Mycophenolic Acid Alters Nucleotide and Lipid Metabolism in an Intestinal Cell Model. *Sci. Rep.* **7**, 45088; doi: 10.1038/srep45088 (2017).

**Publisher's note:** Springer Nature remains neutral with regard to jurisdictional claims in published maps and institutional affiliations.

## Supplementary Material

Supplementary Dataset 1

Supplementary Dataset 2

## Figures and Tables

**Table 1:** Summary of concentration-dependent effects of exogenous guanosine on nucleotide levels and energy charges of LS180 cells exposed to increasing concentrations of MPA.

	0 μM guanosine		200 μM guanosine		1 mM guanosine	
	24 h	72 h	24 h	72 h	24 h	72 h
ATP	↓↓↓	↓↓↓	—	—	↑	—
ADP	—	↓↓↓	—	↓	—	—
AMP	—	↓↓↓	—	↓↓	—	—
GTP	↓↓↓	↓↓↓	↓↓↓	↓↓↓	↓↓↓	↓↓↓
GDP	↓↓↓	↓↓↓	↓↓↓	↓↓↓	↓↓↓	↓↓↓
GMP	↓↓↓	↓↓↓	↓↓↓	↓↓↓	↓↓↓	↓↓↓
UTP	↑↑↑	↑↑↑	—	↑↑↑	↑↑↑	↑↑↑
UDP	↑↑↑	↓↓↓	—	—	—	↑↑
UMP	↑↑↑	↑↑	—	↑	—	—
CTP	↑↑↑	—	—	—	—	↑
CDP	↑↑↑	—	—	—	—	—
CMP	↑↑↑	↑↑	—	—	—	↑
NAD^+^	—	↓↓↓	—	↓↓	—	—
NADP^+^	↑↑	↓↓↓	—	—	—	—
FAD	—	↓↓	—	—	—	—
AEC	—	↑	↓↓	↓↓	—	—
GEC	↓↓↓	↑↑↑	—	—	—	—
UEC	—	—	—	—	—	↑
CEC	—	↓↓↓	—	—	—	—

The table summarizes effects shown in the individual graphs in Supplementary Figs S1 and S2. Most significant changes within one treatment group are indicated in the table. Statistically significant changes (↑↓, number of displayed arrows reflects the most significant change within one treatment group of increasing MPA concentrations *vs*. controls) in LS180 cells treated with different MPA concentrations *vs*. controls are summarized; p,0.05: ↑/↓, p< 0.01: ↑↑/↓↓, p< 0.001: ↑↑↑/↓↓↓. AEC: adenylate energy charge, GEC: guanylate energy charge, UEC: uridylate energy charge, CEC: cytidylate energy charge.

**Table 2:** LS180 cell proteins with changes >20% after exposure of cells to MPA as identified by SILAC in combination with GeLC-MS.

#	Protein name and abbreviation	Uniprot acc. no. Ensemble gene ID	Unique peptides	0.1 μM MPA	5 μM MPA	100 μM MPA	250 μM MPA	r	250 μM MPA
1	Long-chain acyl-CoA synthetase 5 (ACSL5)	Q9ULC5 ENSG00000197142	18	0.83 ± 0.20	1.07 ± 0.08	1.44 ± 0.21^*^	1.94 ± 0.15^**,##^	0.977	193.6
2	UDP glucuronosyltransferase 1A1 (UGT1A1)	P22309 ENSG00000167165	7	1.12 ± 0.25	1.45 ± n.a.	1.55 ± 0.23	1.91 ± 0.29^*^	0.917	191
3	Very long-chain specific acyl-CoA dehydrogenase (VLCAD)	P49748 ENSG00000072778	25	0.94 ± 0.14	1.02 ± 0.11	1.21 ± 0.20	1.83 ± 0.16^**,##, ○^	0.99	183
4	Annexin A1 (ANXA1)	P04083 ENSG00000135046	14	1.17 ± 0.20	1.14 ± 0.11	1.68 ± 0.21	1.75 ± 0.17^*,#^	0.891	174.6
5	Peroxisomal acyl-CoA oxidase 1 (AOX)	Q15067 ENSG00000161533	15	0.88 ± 0.11	1.01 ± 0.09	1.28 ± 0.10^*^	1.75 ± 0.08^***,##, ○○○^	0.992	174.5
6	Fatty acid-binding protein 1 (FABP1)	P07148 ENSG00000163586	4	0.84 ± 0.33	0.91 ± 0.05	1.88 ± 0.31^*,#^	1.70 ± 0.21^*^	0.771	169.9
7	Integrin β-4 (ITGB4)	P16144 ENSG00000132470	46	1.07 ± 0.06	1.27 ± 0.02	1.34 ± 0.04^*^	1.44 ± 0.11^**^	0.836	143.5
8	Guanine nucleotide-binding protein G(I)/G(S)/G(O) subunit γ-12 (GNG12)	Q9UBI6 ENSG00000172380	3	1.01 ± 0.12	1.17 ± 0.03	1.15 ± 0.11	1.35 ± 0.04^*^	0.888	135.4
9	Single-stranded DNA-binding protein 1, mitochondrial (MtSSB)	Q04837 ENSG00000106028	7	0.97 ± 0.10	1.04 ± 0.11	1.02 ± 0.10	1.29 ± 0.07^*^	0.926	128.8
10	Acetyl-CoA acyltransferase (ACAA2)	P42765 ENSG00000167315	8	0.96 ± 0.14	0.89 ± 0.01	1.09 ± 0.11	1.28 ± 0.06^*,##^	0.979	128.4
11	Dihydrolipoamide dehydrogenase (DLD)	P09622 ENSG00000091140	11	0.96 ± 0.04	1.04 ± 0.07	1.09 ± 0.10	1.27 ± 0.07^**^	0.967	127.1
12	Trifunctional enzyme subunit α (TFP)	P40939 ENSG00000084754	21	0.87 ± 0.13	0.94 ± 0.10	1.04 ± 0.07	1.26 ± 0.04^**,#^	0.988	126.4
13	Electron-transfer-flavoprotein (Α-ETF)	P13804 ENSG00000140374	12	0.90 ± 0.02	0.99 ± 0.08	1.09 ± 0.09^*^	1.26 ± 0.04^**,#^	0.974	125.5
14	NAD(P)H dehydrogenase, quinone 1 (NQO1)	P15559 ENSG00000181019	8	1.11 ± 0.04	1.09 ± 0.00	1.25 ± 0.01^*,#^	1.25 ± 0.06^*,#^	0.847	124.6
15	Heat shock 70 kDa protein 9 (Mortalin) (HSPA9)	P38646 ENSG00000113013	25	0.98 ± 0.08	1.02 ± 0.05	1.03 ± 0.01	1.24 ± 0.07^**,#, ○^	0.956	124.5
16	Delta(3,5)-delta(2,4)-dienoyl-CoA isomerase, mitochondrial (ECH1)	Q13011 ENSG00000104823	14	0.91 ± 0.05	0.88 ± 0.07	1.03 ± 0.06	1.22 ± 0.10^*,#^	0.995	122.4
17	Glycerol-3-phosphate dehydro-genase 2, mitochondrial (GPD2)	P43304 ENSG00000115159	22	0.91 ± 0.03	1.04 ± 0.05	1.02 ± 0.10	1.22 ± 0.13^*^	0.886	122.3
18	GTP:AMP phosphotransferase, mitochondrial (Adenylate kinase 3, AK3)	Q9UIJ7 ENSG00000147853	7	0.79 ± 0.13	0.86 ± 0.09	1.01 ± 0.07	1.21 ± 0.14^*^	0.988	121.4
19	Elongation factor T/P43 (EF-Tu)	P49411 ENSG00000178952	18	0.98 ± 0.02	1.00 ± 0.02	0.99 ± 0.01	1.20 ± 0.09^**,#, ○○^	0.918	120.4
20	Succinyl-CoA ligase [ADP/GDP-forming] subunit α (SCS-α)	P53597 ENSG00000163541	3	0.88 ± 0.14	1.06 ± 0.05	1.05 ± 0.07	1.20 ± 0.06^*^	0.842	120.1
21	Polymeric immunoglobulin receptor (PigR)	P01833 ENSG00000162896	13	0.97 ± 0.04	0.78 ± 0.05^*^	0.45 ± 0.05^***,##^	0.35 ± 0.05^***,###^	−0.888	34.8
22	Regenerating islet-derived protein 4 (REG-4)	Q9BYZ8 ENSG00000134193	8	0.86 ± 0.06	0.93 ± 0.15	0.61 ± 0.12	0.48 ± 0.15^*,#^	−0.95	47.7
23	Solute carrier family 12 member 2 (SLC12A2)	P55011 ENSG00000146828	30	0.96 ± 0.05	0.99 ± 0.05	0.63 ± 0.07^**,##^	0.48 ± 0.06^***,###^	−0.952	48
24	Creatine kinase B-type (B-CK)	P12277 ENSG00000166165	16	0.93 ± 0.05	0.74 ± 0.06^*^	0.59 ± 0.06^**^	0.50 ± 0.05^***,#^	−0.969	49.8
25	Cadherin-17 (CDH17)	Q12864 ENSG00000079112	24	0.98 ± 0.04	0.96 ± 0.05^○○^	0.75 ± 0.02^**,##, ○○^	0.63 ± 0.04^***,###, ○^	−0.969	62.7
26	Dihydropyrimidinase-like 2 variant (DPR-2)	Q16555 ENSG00000092964	7	1.13 ± 0.13	0.97 ± 0.24	0.84 ± 0.05	0.63 ± 0.09^*^	−0.946	63.4
27	Stromal cell-derived factor 2-like 1 (SDF2L1)	Q9HCN8 ENSG00000128228	3	1.08 ± 0.02	1.02 ± 0.28	0.83 ± 0.07	0.67 ± 0.06^*^	−0.978	66.5
28	Acetyl-CoA acetyltransferase (ACAT2)	Q9BWD1 ENSG00000120437	8	1.04 ± 0.08	1.00 ± 0.05	0.95 ± 0.08	0.70 ± 0.03^**,##, ○^	−0.978	66.5
29	Tubulin α-4A chain (TUBA4A)	P68366 ENSG00000127824	3	1.12 ± 0.09	0.97 ± 0.03	0.96 ± 0.10	0.74 ± 0.05^**^	−0.926	74
30	UDP-glucose:glycoprotein glucosyltransferse 1 (UGT1)	Q9NYU2 ENSG00000136731	33	0.93 ± 0.03	0.93 ± 0.03	0.89 ± 0.07	0.74 ± 0.08^*^	−0.978	74.4
31	Tubulin α-1C chain (TUBA1C)	Q9BQE3 ENSG00000167553	2	1.13 ± 0.09	0.97 ± 0.01	0.96 ± 0.10	0.75 ± 0.05^**^	−0.909	75.3
32	Sodium/potassium-transporting ATPase subunit β-1 (ATP1B1)	P05026 ENSG00000143153	6	0.92 ± 0.06	1.02 ± 0.00	0.79 ± 0.04 ^##^	0.77 ± 0.05^*,##^	−0.824	77.1
33	Tubulin β chain (TUBB)	P07437 ENSG00000196230	4	1.15 ± 0.11	1.05 ± 0.10	0.98 ± 0.11	0.77 ± 0.07^*^	−0.969	77.1
34	GTP-binding nuclear protein RAN (RAN)	P62826 ENSG00000132341	8	1.08 ± 0.09	0.93 ± 0.01	0.92 ± 0.02^*^	0.79 ± 0.03^**^	−0.867	78.9
35	Tubulin β-4A chain (TUBB4A)	P04350 ENSG00000104833	1	1.13 ± 0.11	1.05 ± 0.07	1.01 ± 0.09	0.79 ± 0.07^*^	−0.966	79.2

The table shows protein numbers (assigned for clarity; increased: proteins #1–20, decreased: proteins #21–35), protein names, abbreviations, Uniprot accession numbers, Ensemble gene IDs, numbers of unique peptides identified, SILAC heavy/light (H/L) ratios for four MPA concentrations, Pearson product-moment correlation coefficients r for the correlation/linear dependence of H/L ratios and MPA concentrations, and the increase in protein levels after exposure to 250 μM MPA compared to a H/L ratio = 1 as expected for controls (=calculation as % control). H/L ratios are given as means ± standard deviations (n = 3). Significance was determined for effects of increasing MPA concentrations using one-way ANOVA combined with Scheffe’s post-hoc test with ^*/#/○^p < 0.05, ^**/##/○○^p < 0.005, ^***/###/○○○^p < 0.001 vs. treatment with 0.1, 5, and 100 μM MPA. *Significance vs. treatment with 0.1 μM MPA, ^#^significance vs. treatment with 5 μM MPA, ^○^significance vs. treatment with 100 μM MPA. Due to a lack of H/L-labeled controls, only treatments of different MPA concentrations were compared among each other.
