# Supplementary material for: The Immunosuppressant Mycophenolic Acid Alters Nucleotide and Lipid Metabolism in an Intestinal Cell Model
Source: Sci Rep. 2017 Mar 22;7:45088. doi: 10.1038/srep45088 (PMC5361167; doi:10.1038/srep45088)
Supplement: Supplementary Dataset 1 [file srep45088-s1.doc]

**The Immunosuppressant Mycophenolic Acid Alters Nucleotide and Lipid Metabolism in an Intestinal Cell Model**

Svenja Heischmann, Monika Dzieciatkowska, Kirk Hansen, Dieter Leibfritz, Uwe Christians

**Supplementary Materials**

# Supplementary Materials and Methods

## Cell culture model

LS180 cells were purchased from the American Type Culture Collection (ATCC, Manassas, VA; CL-187) in passage number 40. Cells were cultured in Dulbecco's Modified Eagle Medium (DMEM)-high glucose (Sigma, St. Louis, MO; DMEM 2964) and supplemented with antibiotics (100 U/mL penicillin and 100 μg/mL streptomycin, Sigma). Cells were supplemented with 10% fetal bovine serum (FetalPlex Animal Serum Complex; Gemini Bio-Products, West Sacramento, CA) and subcultivated every 3-4 days (split ratio 1:3) using an ethylenediaminetetraacetic acid solution (1 L: 9 g NaCl, 0.2 g EDTA, 1 g glucose, 20 mL 1 M KP_i_ pH 7.6 dissolved in 1 L H_2_O, filtered sterile). Cells were cultured at 37 °C in a humidified atmosphere with 5% CO_2_.

## Measurement of high-energy phosphate levels

**Extraction:**

Samples were extracted with 12% perchloric acid [^1^](#_ENREF_1). Lyophilized cell extracts were reconstituted in 500 μL H_2_O. Samples were diluted 1:10 and N^6^-(6-aminohexyl)adenosine 3',5'-diphosphate (6-aminohexyl-ADP, Sigma) at 0.5 μM was added as internal standard. A mix containing all analytes at 0.5 mM concentrations served for preparation of the calibration curve (0.03125-16 μM).

**HPLC-MS analysis:**

HPLC-MS measurements were performed on an API 4000 triple quadrupole (AB Sciex) with adjustments to a method that was previously published by Klawitter et al. [^1^](#_ENREF_1). The instrument was operated in the negative MRM mode with ESI source in combination with an Agilent 1100 HPLC system. Analytes were extracted using a ZORBAX C18, 5 μm (4.6 mm x 5 mm) column (Agilent Technologies) and separated using a Phenomenex Synergy Hydro C18, 3 μm (4.6 mm x 250 mm) column (Phenomenex, Torrance, CA, USA).

The solvents were 2 mM/4 mM dibutylammonium formate (DBAF, mobile phase A) and methanol + 0.1% formic acid (FA, mobile phase B). The gradient used for inline extraction was: 0.0-0.7 min 3% 4 mM DBAF buffer at a flow rate of 1 mL/min, 0.7-0.8 min 3-100% 4 mM DBAF buffer at a flow rate of 1 mL/min, 0.8-14.0 min 100% 4 mM DBAF buffer at a flow rate of 100 μL/min, 14.0-16.0 min 100% 4 mM DBAF buffer at a flow rate of 1 mL/min, 16.0-16.1 min 100-3% 4 mM DBAF buffer at a flow rate of 1 mL/min, 16.1-18.0 min 3% 4 mM DBAF buffer at a flow rate of 1 mL/min for re-equilibration to starting conditions. The gradient used for separation (at a constant flow rate of 700 μL/min) was: 0.0-1.0 min 5% 2 mM DBAF buffer, 1.0-3.0 min 5-20% 2 mM DBAF buffer, 3.0-10.0 min 20-40% 2 mM DBAF buffer, 10.0-13.0 min 40-52% 2 mM DBAF buffer, 13.0-14.0 min 52-80% 2 mM DBAF buffer, 14.0-14.8 min 80-5% 2 mM DBAF buffer, 14.-18.0 min 5% 2 mM DBAF buffer for re-equilibration to starting conditions. Nitrogen was used as drying and collision gas.

The MS parameters were adjusted as follows: ion transition ATP: m/z = 506.3 [M-H]^-^ → 408.2 with 40 msec dwell time, with -83 V DP, -28 V CE, and -13 V CXP; ion transition ADP: m/z = 426.3 [M-H]^-^ → 328.2 with 40 msec dwell time, with -90 V DP, -23 V CE, and -15 V CXP; ion transition AMP: m/z = 346.2 [M-H]^-^ → 79.0 with 40msec dwell time, with -80 V DP, -42 V CE, and -5 V CXP; ion transition GTP: m/z = 522.2 [M-H]^-^ → 424.2 with 80 msec dwell time, with -85 V DP, -30 V CE, and -15 V CXP; ion transition GDP: m/z = 442.2 [M-H]^-^ → 344.2 with 80 msec dwell time, with -85 V DP, -30 V CE, and -15 V CXP; ion transition GMP: m/z = 362.2 [M-H]^-^ → 79 with 80 msec dwell time, with -85 V DP, -42 V CE, and -7 V CXP; ion transition UTP: m/z = 483.2 [M-H]^-^ → 385.3 with 80 msec dwell time, with -90 V DP, -32 V CE, and -14 V CXP; ion transition UDP: m/z = 403.2 [M-H]^-^ → 79.0 with 80 msec dwell time, with -90 V DP, -45 V CE, and -15 V CXP; ion transition UMP: m/z = 323.2 [M-H]^-^ → 79 with 80 msec dwell time, with -90 V DP, -41 V CE, and -6 V CXP; ion transition CTP: m/z = 482.3 [M-H]^-^ → 384.1 with 80 msec dwell time, with -80 V DP, -32 V CE, and -15 V CXP; ion transition CDP: m/z = 402.2 [M-H]^-^ → 304.1 with 100 msec dwell time, with -80 V DP, -28 V CE, and -13 V CXP; ion transition CMP: m/z = 322.1 [M-H]^-^ → 79.0 with 80 msec dwell time, with -80 V DP, -42 V CE, and -8 V CXP; ion transition NAD^+^: m/z = 662.1 [M-H]^-^ → 540.4 with 100 msec dwell time, with -75 V DP, -21 V CE, and -10 V CXP; ion transition NADP^+^: m/z = 742.1 [M-H]^-^ → 620.5 with 120 msec dwell time, with -65 V DP, -22 V CE, and -17 V CXP; ion transition FAD: m/z = 784.0 [M-H]^-^ → 346.1 with 80 msec dwell time, with -140 V DP, -40 V CE, and -13 V CXP; ion transition 6-aminohexyl-ADP: m/z = 525.0 [M-H]^-^ → 233 with 40 msec dwell time, with -90 V DP, -36 V CE, and -19 V CXP. The source temperature was set to 500 ˚C with a capillary voltage of -4200 V and CAD set to 12. The column oven temperature was set to 45 ± 1 ˚C. The injection volume was 100 μL. Total run time was 18 min.

Analyst software (version 1.5.1) was used for quantitation.

## cAMP/cGMP assays

For measurements of cAMP and cGMP in LS180 cell lysates Cyclic AMP/GMP XP Assay Kits (Cell Signaling Technology) were performed according to the manufacturer’s instructions.

## SILAC and GeLC-MS analysis

**SILAC:**

Cells were labeled with either heavy lysine ([U-^13^C_6_]-L-lysine, MW = 152.1259) or light lysine ([^12^C_6_]-L-lysine, MW = 146.1055) according to the manufacturer's instructions (Invitrogen, SILAC Protein Identification and Quantification Kits, manual part no. 25-0841). Cells labeled with heavy and light lysine were incubated with respective MPA concentrations or DMSO (controls), respectively, and 1 mM guanosine (dosed every 24h) without fetal bovine serum. For extraction cells were washed three times with 5 mL PBS prior to removal from plates. Cells were washed (pelleted and resuspended) five times with 3 mL PBS and lysed using 300 μL lysis buffer (Cell Signaling, containing AEBSF). Prior to GeLC-MS experiments incorporation efficiency of heavy lysine was ensured to be >99%. In-solution tryptic digestion was performed on 50 μL of sample following a modified Promega ProteaseMAX Surfactant (Trypsin Enhancer) protocol and samples were analyzed on a LTQ-FT Ultra hybrid mass spectrometer (Thermo Scientific, for digestion and MS analysis please see below).

**1D gel electrophoresis:**

Samples were prepared at 40 μg protein per sample (20 μg of H/L-labeled protein each; bicinchoninic acid protein assay, Pierce Biotechnology) in NuPAGE LDS sample buffer (Invitrogen) yielding a final volume of 20 μL. Samples were heated for 10 min under shaking at 90 ˚C and loaded onto NuPAGE Bis-Tris 4-20% gradient gels (Invitrogen), which were run using MES SDS running buffer and an X-Cell II Mini Gel System (Invitrogen) at 200 V, 120 mA, 25 W per gel for 30 min. BenchMark Protein Ladder (Invitrogen) was used as a protein molecular mass marker. After visualizing proteins using Coomassie Blue (Invitrogen), each lane of the gel was cut into 13 bands of equal size covering the same molecular weight ranges for all samples.

Gel pieces were destained in 200 μL 25 mmol/L ammonium bicarbonate in 50% (vol/vol) ACN for 15 min. 200 μL 100% ACN was applied for 15 min at room temperature. Disulfide bonds were reduced by 10 mM dithiothreitol for 30 min at 65 °C. Cysteine residues were alkylated with 20 mmol/L iodoacetamide in the dark at room temperature for 45 min. Samples were washed with 200 μL of distilled water, then treated with 100 μL ACN. After ACN removal samples were rehydrated in 50 μL 0.01 μg/μL trypsin solution at 4 °C for 30 min and incubated at 37 °C overnight. Samples were acidified with formic acid (final concentration 1%). Peptides were extracted from samples 3 times with 50% ACN containing 1% formic acid and concentrated using a SpeedVac (Thermo Scientific) to a volume of about 18 μL

**HPLC-MS analysis:**

Samples were analyzed on a LTQ-FT Ultra hybrid mass spectrometer (Thermo Scientific). Peptide desalting and separation was achieved using a dual capillary/nano pump HPLC system (Agilent 1200, Palo Alto, CA). On this system, 8 μL of sample were loaded onto a ZORBAX 300SB-C18, 5 μm (5 x 0.3 mm) column (Agilent Technologies) to extract analytes and washed with 5% ACN + 0.1% FA at a flow rate of 15 μL/min for 5 min. The extraction column was mounted in line with the nano pump at a flow rate of 350 nL/min. An 85 min gradient from 8% ACN to 40% ACN was used to separate the peptides. The column was made from an in-house-pulled 360/100 nm (outer/inner diameter) fused silica capillary packed with Jupiter C18 resin (Phenomenex, Torrance, CA). The column was kept at 40 ˚C using an in-house-built column heater. Data acquisition was performed using Xcalibur software (version 2.0.6). The HPLC runs were monitored in positive ion mode by sequentially recording survey MS scans (m/z = 400-2000), in the ion cyclotron resonance cell, while three MS^2^ were obtained in the ion trap via CID for the most intense ions. After two acquisitions of a given ion within 45 s the ion was excluded for 150 s. As an example, Supplementary Figure 4 shows SILAC GeLC-MS data for VIILMDPFDDDLK peptide, one of the unique peptides that served to identify ACSL5.

**Database searching, protein identification, and online analysis tools**:

MS/MS spectra were extracted from raw data files and converted to .mgf files using an in-house script. Mascot (Matrix Science Inc., London, UK; version 2.2) was used for database searches against the human subset SwissProt database of the extracted MS/MS data. Peptide tolerance was set to ± 15 ppm with MS/MS tolerance set to ± 0.6 Da. Trypsin specificity allowed for one missed cleavage. Carbamidomethylation on cysteine residues was defined as fixed modification, methionine oxidation and N-terminal acetylation were defined as variable modifications.

Scaffold Proteome software was used to validate MS/MS-based peptide and protein identifications. Peptide identifications were accepted, if established at greater than 95% probability as specified by the Peptide Prophet algorithm. Peptide identifications were accepted, it established at greater than 99% probability and containing at least two identified unique peptides.

The MaxQuant Software package [^2^](#_ENREF_2) (Max Planck Institute of Biochemistry, Martinsried, Germany, version 1.2.2.5) in combination with the human subset of the FASTA database (version 3.87) was used to further process GeLC-MS data. Peptide identification and quantification parameters were: Peptide false discovery rate (FDR): 0.01, Site FDR: 0.01, Max. peptide posterior error probability: 1, Min. peptides: 2, Min. razor + unique peptides: 1, Min. unique peptides: 2 (at greater than 99% probability). Only unmodified peptides were used and carbamidomethylation on cysteine residues was defined as fixed modification, methionine oxidation and N-terminal acetylation were defined as variable modifications. Protein FDR: 0.01, Min peptide length: 6 amino acids. Parameters for MS/MS & sequences were set to ± 15 ppm and ± 0.6 Da for MS/MS tolerance. As fixed modification “Carbamidomethyl (C)” was used.

Protein ID hits without sufficient amounts of data for statistical analysis were discarded and remaining IDs were filtered for changes in H/L rations of >20% between H/L=1 (ideal control value) vs. H/L (250μm MPA-treated/-untreated cell lysates) = 1.2 (for upregulated proteins) or 0.8 (for downregulated proteins) and proteins that passed the threshold were subjected to statistical analysis.

The Database for Annotation, Visualization, and Integrated Discovery (DAVID) facilitates functional annotation and systematic analysis of a given list of genes/proteins and consists of several tools. For analysis of GeLC-MS data the following tools were used: Functional Annotation Clustering (Tool 1A), Functional Annotation Chart (1B), Functional Annotation Table (1C), and Functional Annotation Chart (2).

For comparison of our data to currently available proteomics data on MPA toxicity in rat models [^5-7^](#_ENREF_5) and other human cell culture models using cells of non-cancerous origin DAVID’s tool 1A and 1B (pathway enrichment analysis using KEGG ) were used. Identifiers of individual proteins listed in the named publications were reassigned prior to DAVID analyses as original identifiers did not represent the correct species.

Default settings as of which databases to search were used but the output was modified for further analysis as follows: Tool 1A: Classification stringency: Kappa similarity: Similarity term overlap = 4, Similarity threshold = 1.00; Classification: Initial group membership = 4, Final group membership = 4, Multiple linkage threshold = 0.5, Enrichment Thresholds: EASE score = 0.05. Tool 1B: Count = custom, EASE score = 0.05. Tool 1C: default settings. Tool 2: Kappa similarity: Similarity term overlap = 4, Similarity threshold = 0.3; Classification: Initial group membership = 4, Final group membership = 3, Multiple linkage threshold = 0.5.

Proteins of pathways that were significantly affected by MPA-treatment (EASE score < 0.05) as identified by pathway analysis using DAVID and KEGG (Tool 1B) were subjected to Pathway Palette analysis [^12^](#_ENREF_12), a software for peptide-, protein-, and pathway-oriented analysis of MS data. Protein-protein interaction networks were generated based on the BioGRID Interaction Database [^13^](#_ENREF_13) and the Human Protein Reference Database (HPRD) [^14^](#_ENREF_14), respectively. Additional potentially affected proteins were identified based on the number of interactions with differentially expressed proteins.

## Cross-reference with published data on proteome alterations in rat models and other human cell lines of non-cancerous origin

DAVID was used for comparison/validation of our data with previously published data on MPA-induced proteome alterations gained through the use of rat models [^5-7^](#_ENREF_5) and human cell lines of non-cancerous origin . For comparison of the LS180 cell model (human colon cancer) with a common rat model (Wistar rats), proteins/genes from the three available publications on this topic (kidney tissue of rats that were treated with mycophenolate mofetil (MMF formulation of MPA) [^5^](#_ENREF_5), liver and colon tissue of MMF-treated rats [^6^](#_ENREF_6), analysis of gene expression (complementary DNA (cDNA) microarray analysis) in liver and gut of MMF-treated rats [^7^](#_ENREF_7)) were pooled (Supplementary Table 1). Gene IDs were converted to protein IDs (Uniprot accession numbers) using DAVID's Gene ID Conversion tool. For comparison with other human cell lines (HEK-293, CCRF-CEM; differential proteome analysis) proteins/genes from the two available publications were combined [^26^](#_ENREF_26), [^27^](#_ENREF_27) (Supplementary Table 2).

## Western blot analysis

Cells were extracted with Cell Lysis Buffer (Cell Signaling, 9803; containing . 4-(2-aminoethyl)benzenesulfonyl fluoride hydrochloride (AEBSF) as protease inhibitor, Sigma,). Protein content was determined by the bicinchoninic acid protein assay (Pierce Biotechnology). Extracts were prepared at a concentration of 100 μg / 15 μL in 1X Laemmli sample buffer (Bio-Rad). Lysates were boiled for 10 min and 100 μg protein (15 μL) per well were loaded onto 26-well TCR-gels (Bio-Rad, Criterion Tris-HCl precast gels, no. 345-0034). 5 μL Precision Plus Protein Dual Color Standard (Bio-Rad) and 2 μL Biotinylated Protein Ladder (Cell Signaling) were used per gel. Gelelectrophoresis was performed at 170 V. Proteins were transferred onto polyvinylidene fluoride membranes at 90 V. Membranes were washed in Tris-buffered saline (TBS, 1 L 1X TBS: 8 g NaCl, 0.2 g KCl, 3 g Tris-base dissolved in 1 L H_2_O, pH 7.4) with 0.1% Tween-20 (TTBS) for 15 min and blocked in 5% milk in TTBS for 1 h. Membranes were incubated with the respective first antibodies (as specified below) in 5% milk in TTBS for 2 h and washed 3 times for 10 min in TTBS. It was incubated with the respective horseradish peroxidase (HRP)-coupled secondary antibodies (listed below) in 5% milk in TTBS for 1 h and washed 3 times for 10 min with TTBS. Membranes were incubated with chemiluminescent solution (Bio-Rad, Immun-Star HRP Luminol/Enhancer and Immun-Star HRP Peroxidase Buffer mixed 1:1) for 5 min. Films were exposed to the chemiluminescent membranes for varying time periods and developed using a Kodak Image Station 440CF. Band intensities were determined using 1D Image Station software (Kodak). Bands were compared within gel and reported as arbitrary units/% of controls.

The following primary antibodies and dilutions were used: Anti-ACSL5 (antibody 1): Sigma, WH0051703M1, Ms, 1:1000; Anti-ACSL5 (antibody 2): Abcam, ab104892, Rb, 1:1000; Anti-ANXA1: Cell Signaling, 3299, Rb, 1:1000; Anti-SLC12A2, Sigma, AV43805, Rb, 1:1000, Anti-PIgR: Abcam, ab91269, Rb, 1:1000, Anti-REG-4: Abcam, ab89917, Ms, 1:1000; Anti-UBC: Cell Signaling, 3933, Rb, 1:1000; Anti-ZO-1: Cell Signaling, 5406, Rb, 1:1000; Anti-14-3-3 θ: Cell Signaling, 9638, Rb, 1:1000.

The following secondary antibodies and dilutions were used: Anti-Ms: Cell Signaling, 7076, Horse, 1:2000; Anti-Rb: Cell Signaling, 7074, Goat, 1:2000.

## NMR analysis

**Extraction:**

NMR experiments were conducted accorging to Zwingmann et al. [^15^](#_ENREF_15). Approximately 10^6^ cells were used per sample. Cells were extracted with 12% perchloric acid [^1^](#_ENREF_1). Lyophilized protein-lipid samples were reconstituted in 900 μL CDCl_3_/CD_3_OD 2:1, transferred to glass tubes, and centrifuged (20 min, 1450 g, 20 °C). Supernatants were transferred to NMR tubes. Metabolite concentrations were normalized to cell wet weight and determined after perchloric acid extraction.

**Acquisition and processing parameters of NMR spectra:**

NMR spectra were recorded on a Bruker DRX 600 spectrometer (Bremen, Germany) equipped with a 5-mm HCN probe (^1^H spectra of hydrophilic cell extracts and lipids and 2D spectra) or a 5-mm ^1^H/^13^C dual probe (^13^C spectra) at frequencies of 600 MHz (^1^H) and 150 MHz (^13^C). Spectra were recorded using established pulse sequences with water saturation independent of d1 time (d1 = 5*T_1_).

Spectra were processed using MestRe-C software (version 4.9.9.9). Zero-filling to 32k was applied to all spectra. For ^1^H NMR spectra a gaussian apodisiation function (1-2 Hz) was used.

**^1^H-^13^C HSQC NMR parameters:**

Several ^1^H-^13^C HSQC NMR spectra of LS180 hydrophilic cell extracts and lipid extracts were recorded for signal assignment in 1D NMR spectra.

The settings for the recording of ^1^H-^13^C HSQC NMR spectra of lipophilic cell extracts were as follows: NMR probe: 5-mm HCN inverse; Temperature: 300 K; Spectral width F2: 6009.62 Hz, 10.01 ppm; Spectral width F1: 24154.59 Hz, 160 ppm; Number of scans: 32.

The resolution in F1 and the number of scans was defined by acquisition time, metabolite concentration, and desired resolution. 90˚ pulses were determined for each sample individually. Representative values are listed.

**^1^H NMR parameters:**

Parameters for the recording of ^1^H NMR spectra of lipophilic cell extracts were as follows: NMR probe: 5-mm HCN inverse; Temperature: 300 K; Spectral width: 9.50 Hz, 150 ppm; Number of scans: 300; Flip angle: 40˚; Repetition time: 17.2 s; Data size: 16k.

Highly abundant compounds were integrated and quantified after confirmation of signal assignment by 2D NMR. From ^1^H NMR spectra of lipid extracts of MPA-treated LS180 cells, the amounts of established lipid, fatty acid, and other carbon positions were determined, i.e. cholesterol/cholesterolester (Chol, C19 position), fatty acids (F_α_, F_β_, F_ω_, F(CH_2_)_x_+_Fω-1_) Phosphatidylcholine (PtdCho α/β), trimethyl ammonium compounds/choline head groups (N^+^(CH_3_)_3_), diacylglycerols (DAG β), triacylglycerols (TAG β), carbon atoms at a double bond (FΔ-1, monounsaturated fatty acids (MUFA)), polyunsaturated fatty acids (FΔ-CH2-Δ, PUFA), and total number of double bonds (TBD, MUFA + PUFA). Representative examples of ^1^H-^13^C HSQC spectra for signal assignment and ^1^H spectra for each condition are shown in Figure 3, Panel a, b, and c, respectively

**Quantification:**

For ^1^H NMR spectra of lipid extracts, a TSP capillary of known TSP concentration was used as standard (dissolved in CDCl_3_/CD_3_OD 2:1, Sigma). For calibration of the NMR spectra the TSP signal was set to 0.00 ppm.

Due to varying chain lengths of lipids and overlaps of signals an absolute quantification of lipophilic compounds was not possible and lipophilic compounds were quantified as % of controls only. As cholesterol signals overlapped with multiple other signals in lipid spectra (Figure 2, panel a and b) respective compounds were corrected according to the number of protons contributing to the respective cholesterol signals. Values calculated from the isolated cholesterol C18 signal served as correction factors.

**Identification:**

^1^H-^13^C HSQC NMR spectra: Compounds were identified using online databases .

## Reagents

Solvents used for extractions and LC/MS runs were of LC/MS grade and purchased from J.T. Baker (Central City, Pennsylvania) or Thermo Scientific (Fair Lawn, New Jersey). Other consumables were purchased from Sigma or Fisher Scientific unless otherwise specified in the text.

## Statistics

SPSS software (version 19 and 21) was used for statistical analysis. Prior to statistical tests, outliers were removed. One-way analysis of variance (ANOVA) was used for comparison of more than two groups. Scheffe's *post-hoc* test was used to test for significant changes within groups. Data are given as mean ± standard deviation. Differences were considered statistically significant at p<0.05 (*), p<0.01 (**), and p<0.001 (***).

**Supplementary Figures and Tables**


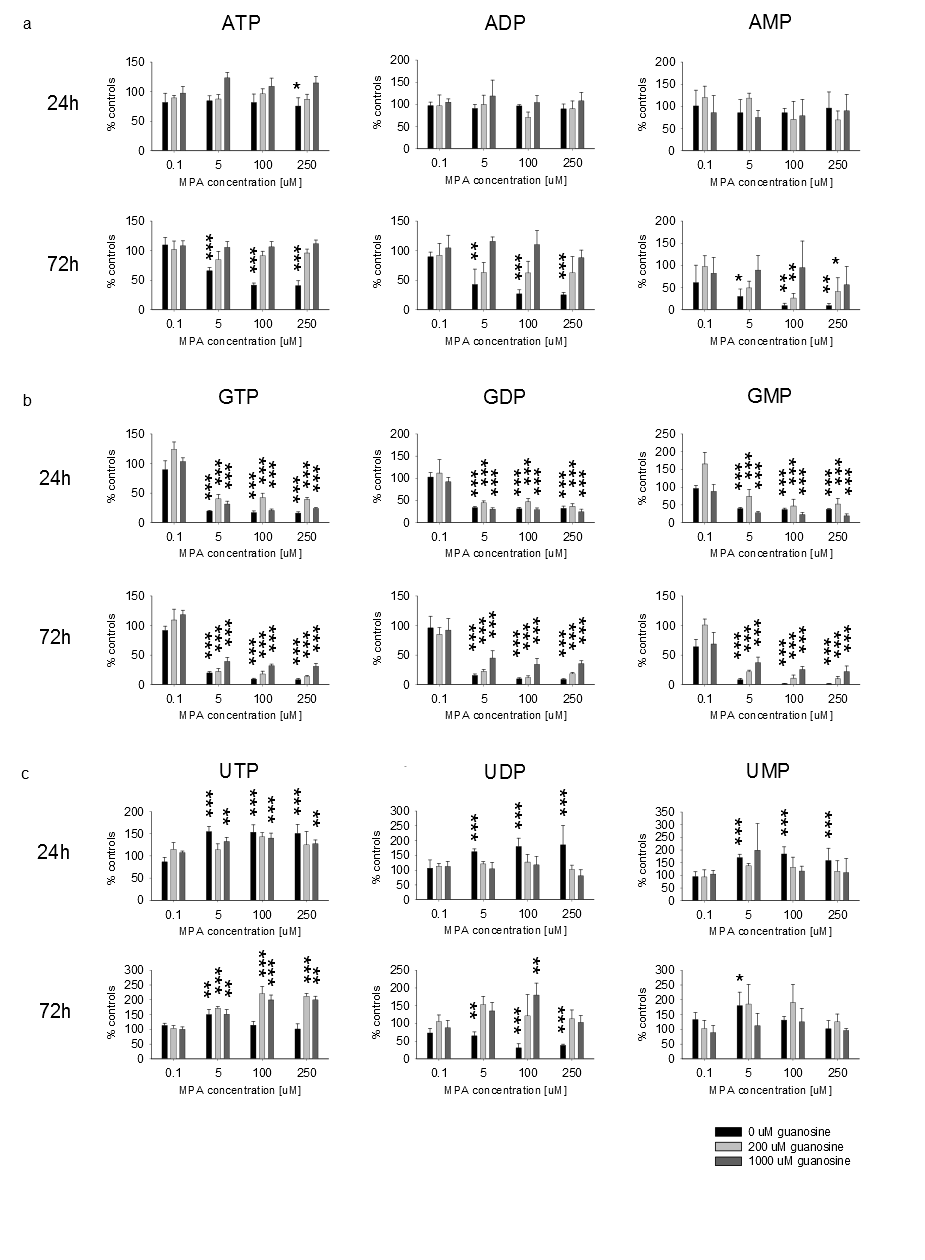


**Supplementary Figure 1: Nucleotide levels in LS180 cells after exposure to different concentrations of MPA and guanosine for 24 and 72 h (dosed every 24 h).** (a) Levels of adenosine nucleotides. (b) Levels of guanosine nucleotides. (c) Levels of uridine nucleotides. Values are given as means of % of control (n = 4). Significance was determined using one-way ANOVA combined with Scheffe’s *post-hoc* test with *: p<0.05, **: P<0.01, ***: p<0.001 *vs.* controls.


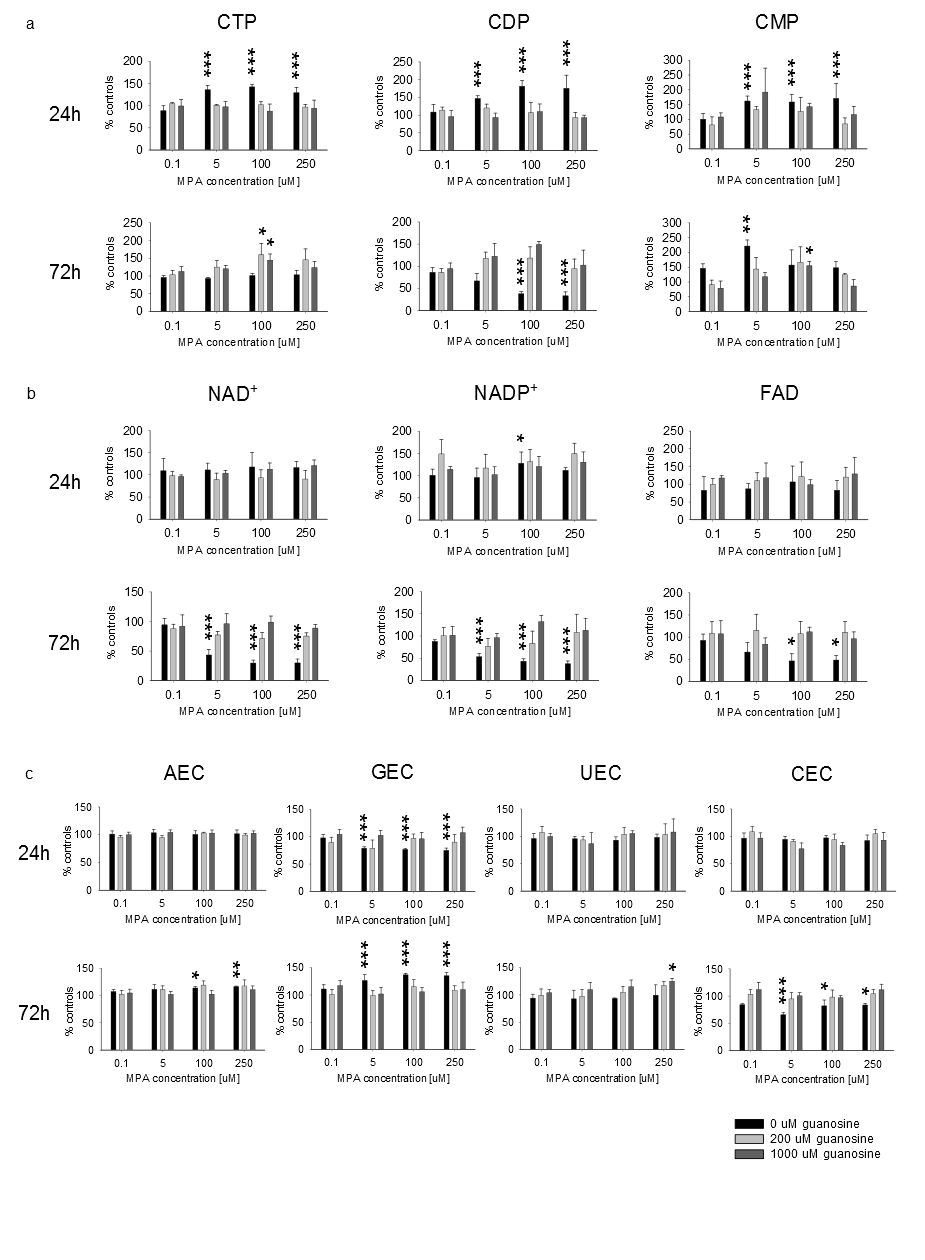


**Supplementary Figure 2: Nucleotide and cofactor levels in LS180 cells after exposure to different concentrations of MPA and guanosine for 24 and 72 h (dosed every 24 h).** (a) Levels of cytidine nucleotides. (b) Levels of the cofactors NAD^+^, NADP^+^, and FAD. (c) Nucleotide energy charges. Values are given as means of % of controls (n = 4). Significance was determined using one-way ANOVA combined with Scheffe’s *post-hoc* test with *: p<0.05, **: P<0.01, ***: p<0.001 *vs.* control.

**
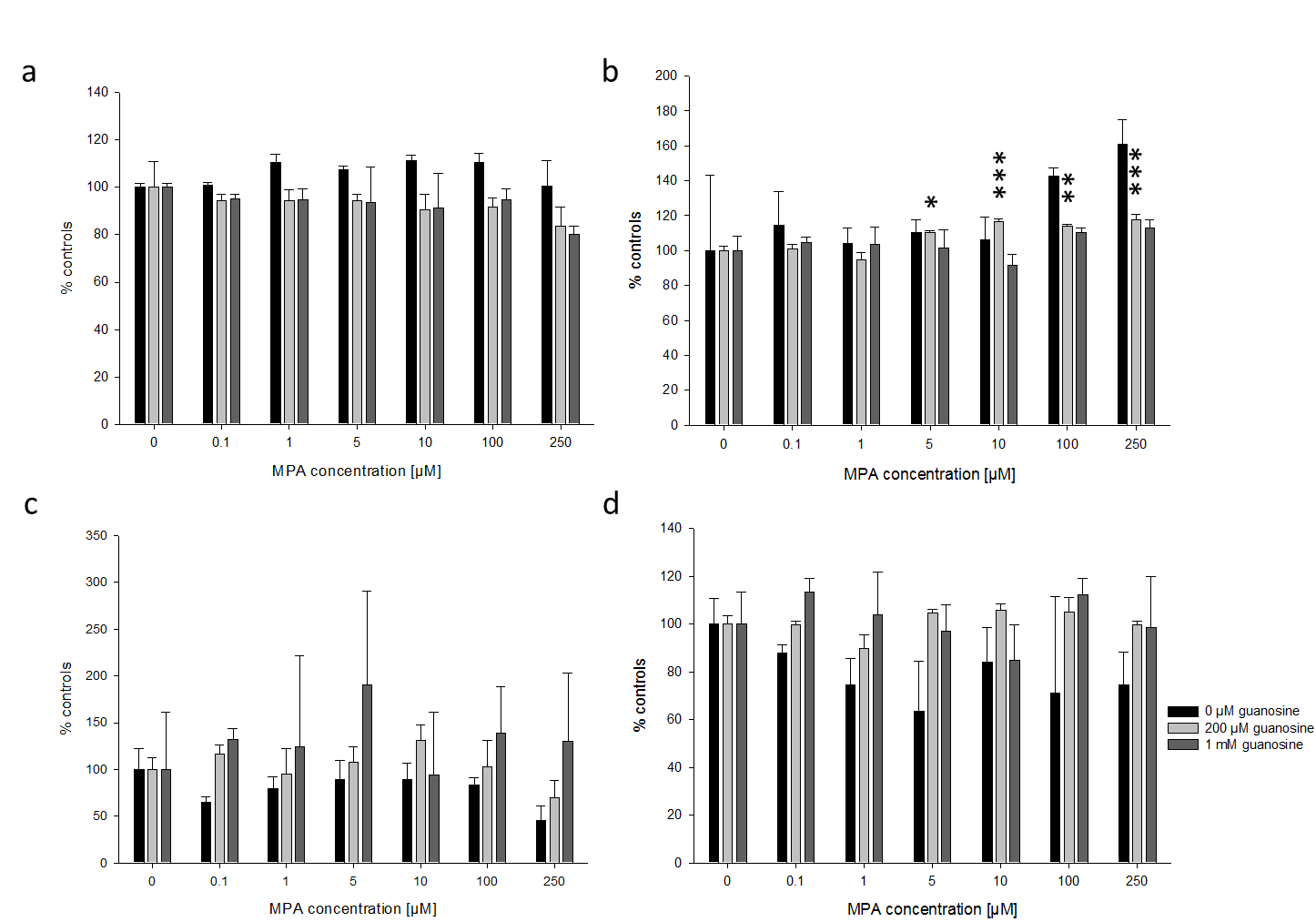
**

**Supplementary Figure 3: Levels of cyclic adenosine and guanosine monophosphate in LS180 cells after exposure to MPA and guanosine for 24 h and 72 h.** (a) Cyclic adenosine monophosphate after 24 h, (b) Cyclic adenosine monophosphate after 72 h. (c) Cyclic guanosine monophosphate after 24 h. (d) Cyclic guanosine monophosphate after 72 h. Values are given as means of % of controls (N = 3). Significance was determined using one-way ANOVA combined with Scheffe’s *post-hoc* test with *: p<0.05, **: P<0.01, ***: p<0.001 *vs.* controls.

**Supplementary Table 1: Proteins/genes affected by MPA treatment in rats.** Proteins/genes are listed with their abbreviations, Uniprot identifiers, and references.

**Supplementary Table 2: Proteins/genes affected by MPA treatment in HEK-293 and CCRF-CEM cells.** Proteins/genes are listed with their abbreviations, Uniprot identifiers, and references.

**
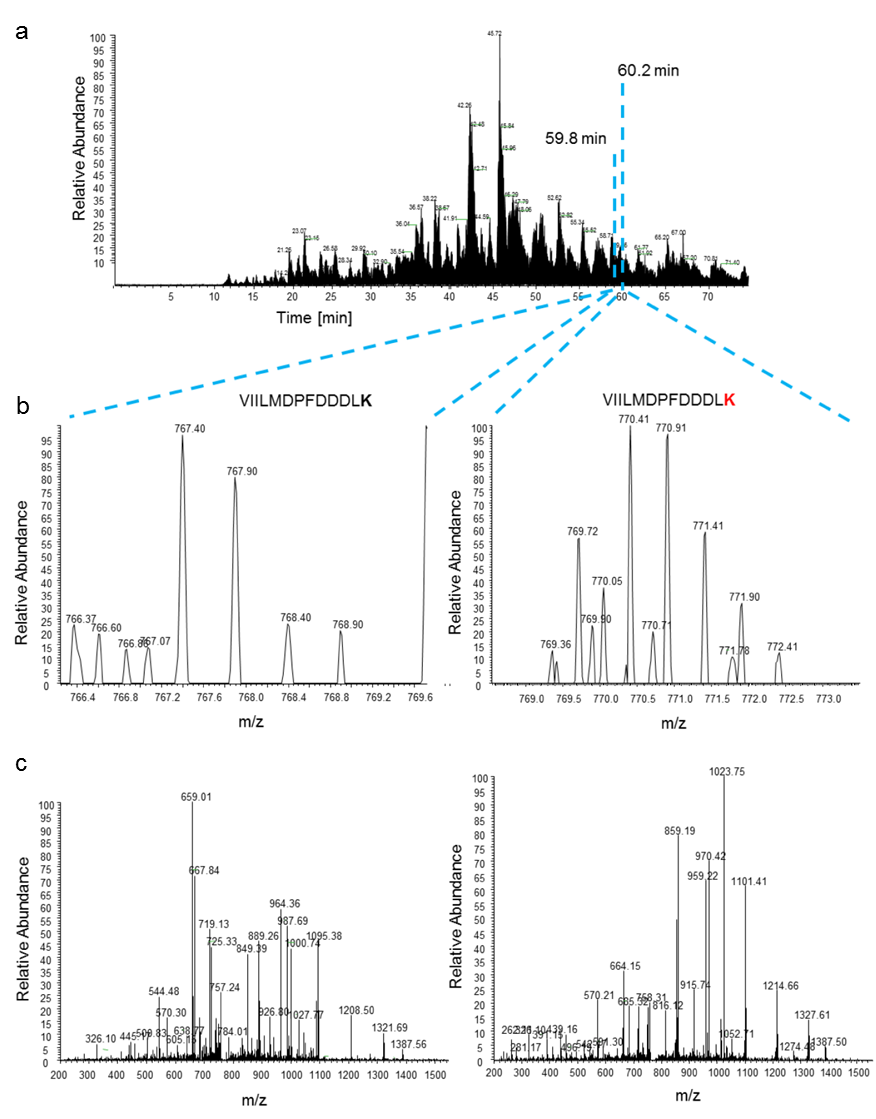
**

**Supplementary Figure 4: HPLC-MS data for VIILMDPFDDDLK peptide, one of the unique peptides that served to identify ACSL5.** (a) Total ion chromatogram. (b) SILAC pair at m/z 767.4 and 770.41 corresponds to the doubly-charged VIILMDPFDDDLK peptide containing one lysine (heavy lysine labeled in red in amino acid sequence). (c) MS/MS spectra of m/z 767.4 and 770.41.

**Supplementary Table 9: Additional proteins identified by Pathway Palette analysis.** Protein numbers (assigned for clarity; using BioGRID (# 1-9) and HPRD (# 10-19, 6*, 9*) databases), protein names, protein and gene (italic) abbreviations, Uniprot accession numbers, the number of interactions identified by Pathway palette analysis as shown in Figure 1 are listed. Only proteins with > 3 interactions are listed. *: previously identified using the BioGRID database and listed in table.


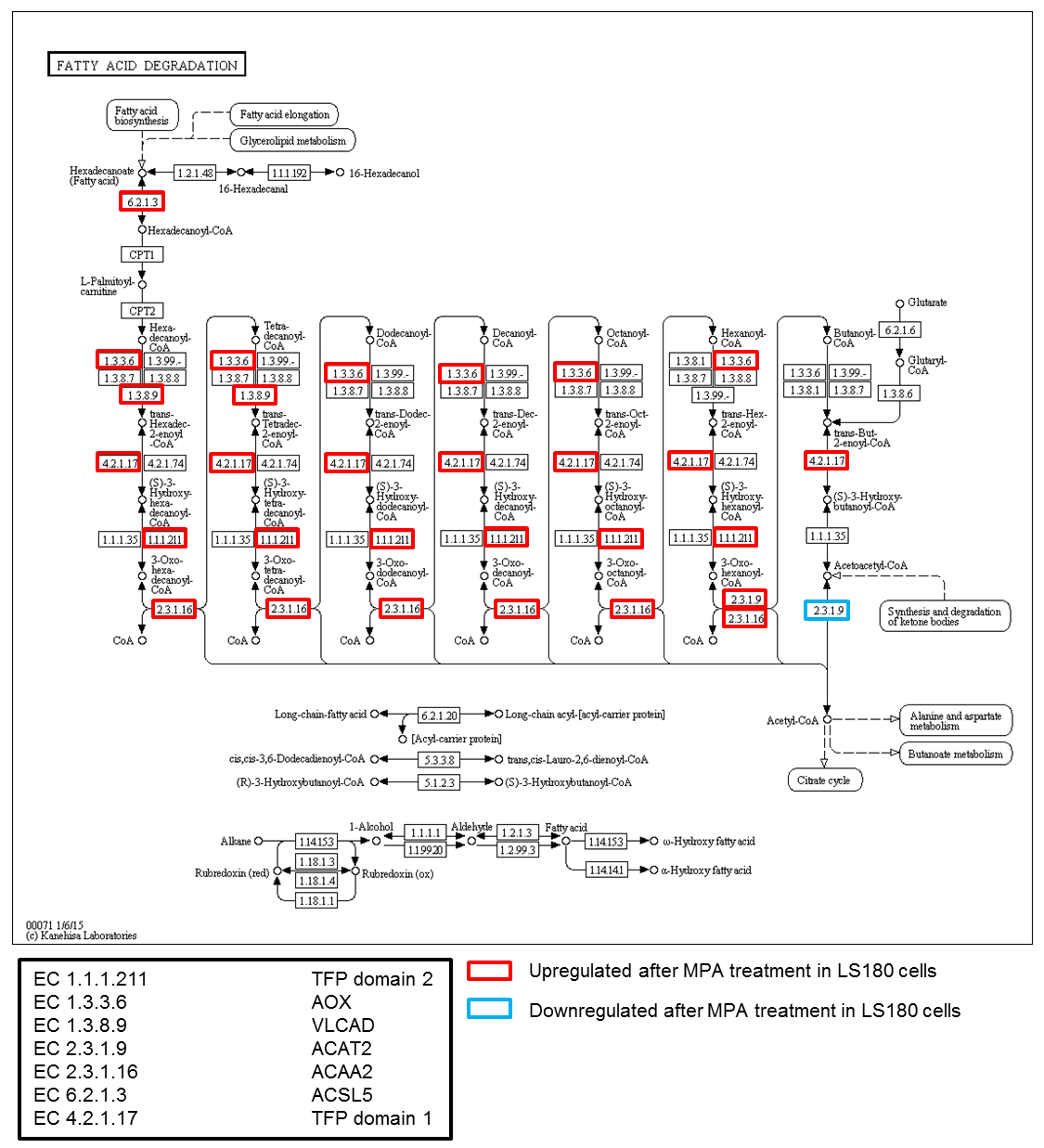


**Supplementary Figure 5: KEGG pathway map 0007: Fatty acid degradation.** EC numbers of proteins significantly affected by MPA treatment in LS180 cells are marked with red (upregulated) and blue (downregulated) boxes.

**
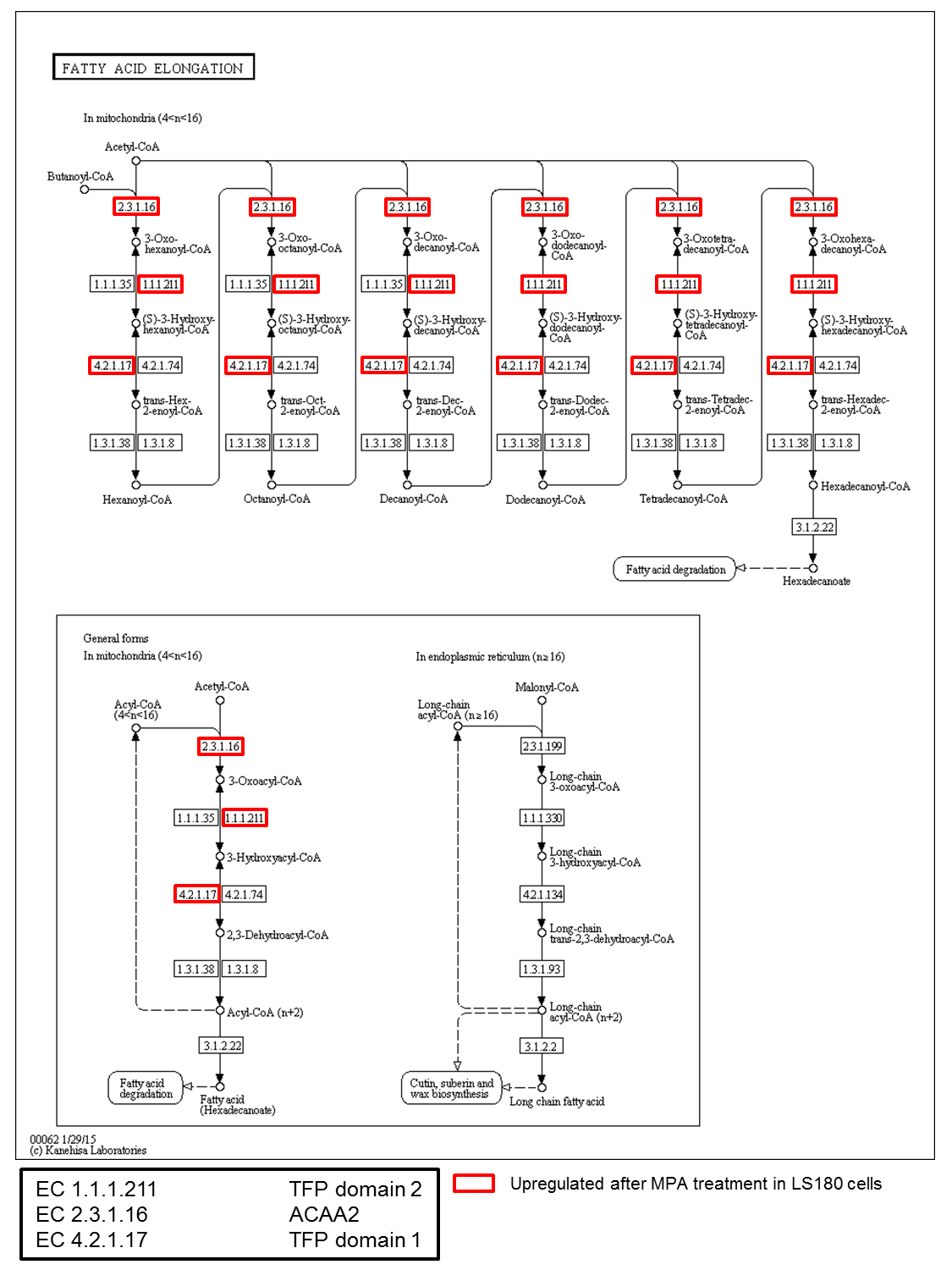
**

**Supplementary Figure 6: KEGG pathway map 00062: Fatty acid elongation.** EC numbers of proteins significantly affected by MPA treatment in LS180 cells are marked with red (upregulated) boxes.

**References (Supplementary Materials)**
